# Valorization of Recycled Tire Rubber for 3D Printing of ABS- and TPO-Based Composites

**DOI:** 10.3390/ma14195889

**Published:** 2021-10-08

**Authors:** Fouad Laoutid, Soumaya Lafqir, Antoniya Toncheva, Philippe Dubois

**Affiliations:** Polymeric and Composite Materials Unit, Materia Nova Research Center, University of Mons UMONS, Nicolas Copernic 3, 7000 Mons, Belgium; soumaya.lafqir@materianova.be (S.L.); antoniya.toncheva@materianova.be (A.T.); philippe.dubois@umons.ac.be (P.D.)

**Keywords:** tire rubber recycling, polymer composites, additive manufacturing, fused deposition modeling

## Abstract

Vulcanized and devulcanized ground tire rubber microparticles have been used as a minor phase in acrylonitrile butadiene styrene copolymer (ABS) and thermoplastic polyolefins (TPO) for the development of materials with desired functionalities by 3D printing. These polymers have been selected because they (*i*) present part of the plastic waste generated by the automotive industry and (*ii*) have totally different properties (ABS for its stiffness and robustness and TPO for its softness and ductility). The study aims to improve the circular economy of the automotive industry by proposing a promising route for recycling the generated tire rubber waste. In this respect, emergent technology for plastic processing such as 3D printing is used, as part of the additive manufacturing technologies for the prolongated end of life of recycled plastics originated from automotive waste such as ABS and TPO. The obtained results revealed that (*i*) the composites are suitable for successful filament production with desired composition and diameter required for successful 3D printing by fused deposition modeling, and that (*ii*) the optimization of the composition of the blends allows the production of materials with interesting mechanical performances. Indeed, some of the investigated ABS-recycled rubber tire blends exhibit high impact properties as TPO-based composites do, which in addition exhibits elongation at break higher than 500% and good compression properties, accompanied with good shape recovery ratio after compression.

## 1. Introduction

Faced with the environmental emergencies requiring the reduction of plastic waste, especially materials such as single-use plastics or used tire rubber, the necessity of finding new solutions for fast recovery of wastes is becoming an absolute and urgent action to be taken.

Tire rubber waste could be valorized as a cost reduction additive into different materials, such as concrete [1], asphalt [2], and cement [3], but also in polymers [4]. Several studies and reviews [5,6,7] have already evidenced the interest and limitations of the incorporation of post-consumer tire waste as an additive into various polymeric materials. Different parameters, such as the nature of the polymeric matrix, the tire rubber content and composition, and the effect of the rubber particle size and dispersion state, have been reported as key factors directly affecting the mechanical properties of resulting composites. To date, a great range of data have been acquired on materials properties prepared mainly by conventional polymer melt processing technologies, such as extrusion and injection/compression molding [5,6,7].

In the present study, we focused our attention on the development of polymeric composite materials, containing tire rubber waste, by fused deposition modeling (FDM) 3D printing technology, as a promising approach for recycled used tire recovery, thus conferring new application fields of the printed composites. FDM 3D printing process is based on the construction of items through successive layer deposition of molten materials, deposited by a mobile extruder fed by a plastic filament [8,9]. In fact, additive manufacturing (AM) is in constant increase in many sectors, and FDM technology has become even more accessible, as it has been widely popularized by Fab labs, thus decreasing the price of 3D printers [10]. As part of a common social and economic concern, the focus of this study is the evaluation of recycled tire rubber waste as an additive to produce thermoplastic composite materials processed by FDM 3D printing. For this purpose, the effect of different parameters was studied: (*i*) the nature of the polymeric matrix, (*ii*) the nature of the recycled rubber, and (*iii*) the impact of the interfacial adhesion between polymeric and rubber phases onto the functional properties of the resulting 3D printed materials, in comparison with the performances of similar in composition materials obtained by conventional injection-compression/molding.

Acrylonitrile-butadiene-styrene copolymer (ABS) and thermoplastic polyolefin (TPO) have been chosen to serve as host polymer matrices in our study and this for several reasons: (*i*) these polymers are widely used and adapted for FDM 3D printing; (*ii*) they present totally different and contrasting mechanical properties—ABS is known for its stiffness and robustness and TPO for its softness and ductility properties; (*iii*) and they represent a substantial part of the current plastic waste, e.g., generated by the automotive industry [11,12]. In addition, the present study also aims at the ambitious goal of improving the circular economy of the automotive industry. In this context, a promising route for the reuse of the tire rubber waste is proposed, while applying emergent AM technologies for ABS and TPO processing, as these plastics are already actively studied as commercial plastics for 3D printing and are found to be a significant part of the recycled plastics originated from automotive waste.

Two types of recycled tire rubbers, presenting two different chemical forms, i.e., (*i*) vulcanized (GTR) and (*ii*) devulcanized (DevGTR), were used. The most important difference between them is related to their ability to deform and flow during melt processing. In fact, vulcanized rubber particles as tridimensional covalent networks could not melt during the melt processing, and subsequently the size of rubber phases in the final composites depends strongly on the size of the initially incorporated vulcanized rubber particles. Moreover, vulcanized rubber domains are not able to achieve a sufficient degree of chain entanglement with the polymer matrix, which create the desired level of strong physical bonding. In contrast, devulcanized phase can deform during melt processing and present more entanglement potential with the polymeric chains of the host matrix. Here, we studied the impact of the incorporation of the soft phase (recycled tire rubber) in both rigid (ABS) and flexible (TPO) matrices, onto the thermal, morphological, and mechanical properties of the final materials.

As a final touch, the effect of a compatibilizing agent on the blend morphology and mechanical properties was also investigated. Taking into account the composition of both polymeric matrices (ABS and TPO) and recycled rubber tire, which contains a complex mixture of different elastomers (mainly polyisoprene, polybutadiene, and styrene-butadiene rubber), filled with different additives (such as carbon black, zinc oxide, oil, textile, sulfur, vulcanization agents and antioxidants), we decided to use a non-functionalized styrene-ethylene/butylene-styrene block copolymer (SEBS) as a compatibilizing agent. Indeed, SEBS has been already reported to act as efficient compatibilizer agents in several blends. Oliphant et al. [13] demonstrated the efficiency of SEBS as a compatibilizer in polyethylene and polypropylene filled with GTR particles, thus significantly enhancing the mechanical properties of compatibilized blends. It has been also demonstrated to increase the interfacial adhesion and morphology of polyolefin (PP)/ABS blends and the overall mechanical properties [14]. Similar behavior was observed by Kong et al. [15] in polypropylene/polystyrene blends compatibilized with styrene-ethylene/propylene-styrene (SEPS). The compatibilizing effect of SEBS in all these systems has been explained by interactions between similar chemical groups, i.e., styrene contained in both SEBS and other phases (GTR, ABS, and PS) as well as to the physical entanglement between ethylene/butylene-based segments with polyolefin chains (PE and PP).

SEBS appears clearly as a suitable candidate for compatibilizing tire rubber particles with both TPO and ABS owing to olefin and styrene segments present, respectively, in both matrices. Moreover, non-functionalized SEBS presents an interesting advantage from an economic point of view since it is relatively cheaper than SEBS functionalized with anhydride maleic (SEBSgMA). This point is very important for developing cost-effective blends and promoting the development of recycled materials. Different polymer/tire rubber blends were obtained by melt processing, using an internal mixer. The obtained composites were thus used to produce filaments suitable for FDM-3D printing and for the sake of comparison for the preparation of specimens by injection-compression/molding. Thermal, morphological, and mechanical properties of the obtained specimens were analyzed. This comparative method allowed us to evaluate the difference in the properties induced by the manufacturing process of materials that are similar in composition and 3D printing vs. injection-compression/molding, and it also allowed us to adjust the blend composition to achieve enhanced performance.

## 2. Materials and Methods

### 2.1. Materials

**Figure 1:**
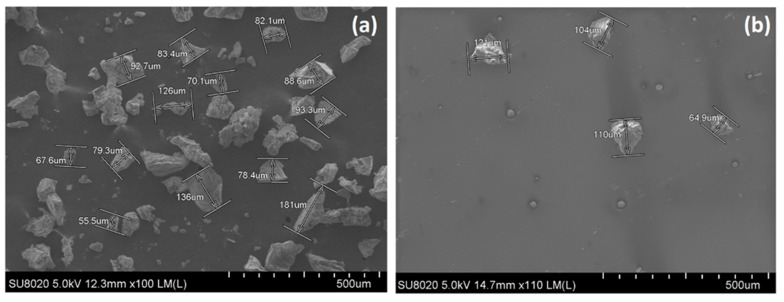
SEM micrograph of (**a**) GTR and (**b**) DevGTR.

FDM 3D printing acrylonitrile-butadiene-styrene copolymer (ABS) and thermoplastic polyolefin elastomer (TPO) were purchased from BBFIL (Heiligenberg-Vallée, France). Two different recycled tire rubber materials, vulcanized (GTR) and devulcanized (DevGTR) tire rubbers, were provided by RubberGreen (Frameries, Belgium) and used to investigate the effect of the chemical nature of the rubber phase on the final properties of the 3D-printed composites. GTR particles were obtained by cryogenic grinding of recycled tire rubber and used as received, while DevGTR was chemically devulcanized by the RubberGreen company and ground in our laboratory under liquid nitrogen, using CryoMill apparatus from Retsch (Haan, Germany) according to the following procedure: 3 cycles of 3 min at 30 Hz with 30 s at 5 Hz between each period. Both GTR and DevGTR presented particle size below 140 µm. (Figure 1). Styrene-ethylene/butylene-styrene (SEBS) block copolymer, KRATON^®^ G1650 E, was purchased from Kraton Corporation (Almere, The Netherlands) and used as compatibilizing agent.

### 2.2. Melt Processing

#### 2.2.1. Polymer Composites Preparation

**Figure 2:**
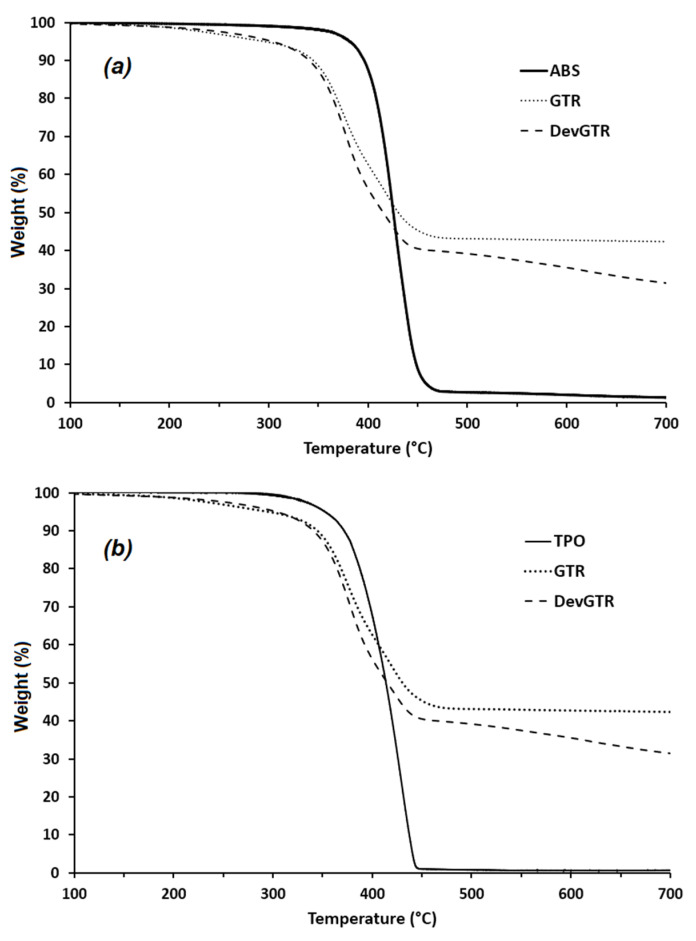
TGA thermograms of vulcanized (GTR), devulcanized (DevGTR) recycled tire rubber powders and ABS (**a**) and TPO (**b**) (under N_2_, 10 °C/min).

ABS-recycled tire composites were prepared in a Brabender internal mixer at 240 °C (3 min mixing at 30 rpm followed by 7 min at 100 rpm), while TPO-recycled tire composites were prepared at 210 °C, following the same working procedure. The melt processing temperatures were selected to ensure that the polymers could melt during the mixing processing, while limiting the thermal degradation of the rubber phase. In fact, the TGA thermograms of the GTR and the DevGTR particles revealed that they present lower thermal stability than both ABS and TPO matrices (Figure 2), but their weight losses, recorded at the melt processing temperatures of ABS and TPO (240 and 210 °C), remained relatively low. In fact, the weight loss of GTR and DevGTR was, respectively, around 2.7% and 3% at 240 °C and 1.6% and 1.4% at 210 °C. In addition, a residue was obtained at the end of the thermal decomposition (from 30 to 40 wt.%) for all the recycled tire rubbers and corresponded to the amount of the mineral fillers that they contained.

#### 2.2.2. Preparation of Specimens for Mechanical Testing

##### By 3D Printing

The prepared composites were processed for the preparation of 3D filaments with diameters of 2.85 mm, using filament maker composer 450 from 3 Devo following the conditions presented in Table 1.

**Figure 3:**
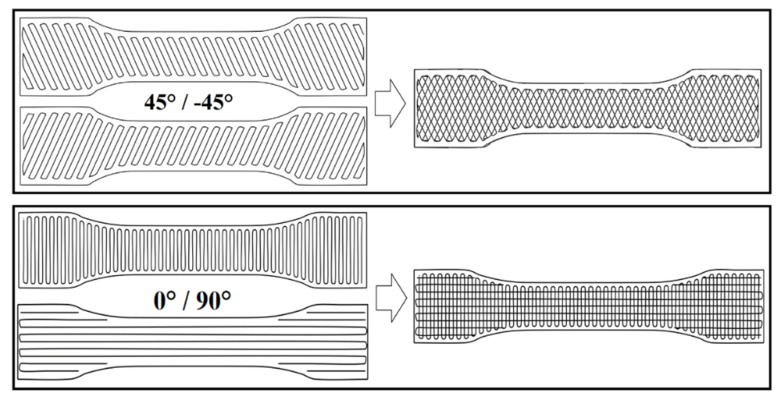
Schematic presentation of the raster angle 45°/−45° and 0°/90°used for the ABS-based samples 3D printing.

The obtained filaments were successfully used for the fabrication of tensile and impact test specimens using FDM-3D printer A4v4 from 3ntr (Oleggio, Italy) with a nozzle diameter of 0.6 mm. TPO filaments were loaded using soft polymer feeder unit (SPFU). The main set of printing parameters used for this step are presented in Table 2, and slicing program Kisslicer Pro was used to allow samples with good 3D printing resolution quality to be obtained. Here, no raft was used, nor any support needed to print the specimens. The orientation of the extruder head when filling inside the contour of the printed part was quantified by the angle of the raster direction. Successive layers may present different printing angles, and it was possible to cross the raster of the consecutive layers. Examples of 0°/90° and crossed 45°/−45° raster direction angles are shown in Figure 3. The infill percentage, corresponding to the amount of the used raw material, was set at 100% to maximize the interaction between filaments. This parameter may range from 0 to 100%, and the lower its value was, the shorter the printing time was and the less material was used in the printed part.

ABS-based specimens of ISO 527 standard (Type IBA with 75 mm length, and 8.86 mm width and 3 mm thickness) were used for tensile characterization, while specimens according to ASTM D638 Type 1 (82.5 mm length, and 9.5 mm width and 3 mm thickness) were used in the case of TPO-based compositions. For both ABS- and TPO-based materials, impact resistance was evaluated according to ASTM D256 standard (samples dimensions: 63.5 mm length, 12.7 mm width, and 3 mm thickness).

##### By Conventional Melt Processing

The mechanical properties of 3D printed materials were compared to those of injected/molded specimens in the case of ABS-based composites and the compressed/molded ones for the TPO-based compositions. ABS-based specimens for the physico-mechanical tests (ATM D638 type II for tensile test and ASTM D256 for impact analysis) were prepared by injection molding using DSM Mini Injection Molding apparatus according to the following procedure: 2 min at 240 °C and injection within a mold at 80 °C under a pressure of 8 bar. TPO specimens (ASTM 638 for the tensile tests and ASTM D256 for impact analysis) for the physico-mechanical tests were cut from polymer films (75 × 75 × 3 mm^3^) prepared by compression molding at 220 °C using an Agila PE20 hydraulic press and following an adapted procedure consisting in: (*i*) preheating of the blend deposited for 3 min between the heated plates, (*ii*) compression of the sample for 3 min at 10 bar, (*iii*) 3 cycled degassing step, (*iv*) sample compression for 3 min at 150 bar, and finally (*v*) a cooling step (5 min at 50 bar).

### 2.3. Characterization

#### 2.3.1. Mechanical Tests

Tensile tests were performed using a Lloyd LR 10 K tensile bench according to ASTM D 638 standard (speed of 10 mm/minute and distance between the clamps of 52.5 mm for ABS-based composites and 50 mm/minute for TPO-based composites and distance between the clamps of 50 mm). For statistical relevance, 5 specimens from each of the samples were preprepared and tested (preconditioning of the specimens for 48 h at 20 ± 1 °C under a relative humidity of 55 ± 5%). The materials impact strength was determined with a Ray-Ran 2500 pendulum impact tester used in Notched Izod mode, following ASTM D256 standard (E = 3.99 J, mass = 0.668 kg and speed = 0.46 m/s). As for the tensile test, 5 specimens of each sample were conditioned and subjected to analysis. The obtained data present average values based on the independent tests. The printed materials compression properties were tested using Lloyd LR 10 K apparatus, equipped with a 1 kN load cell (compression speed of 10 mm/min). The cylindric specimen dimensions were in accordance with the ASTM D695 standard (24.5 mm height and 12.5 mm diameter). The materials shape recovery ratio (Rr) after compression tests was calculated based on Equation 1 (Equation (1)).

Rr = [(l_un_−l_c_)/l_un_] × 100 (1)

where l_un_ is the length of the sample before the compression test (uncompressed sample), l_c_ is the length of the sample after compression strain of 15%. Here, l_c_ is measured 5 min after the compression test, an arbitrary time for the material relaxation and shape recovery. The same printing parameters were applied for the fabrication of the specimens subjected to compression tests.

#### 2.3.2. Scanning Electron Microscopy (SEM)

The materials morphological analysis was performed by SEM Hitachi SU8020 (100 V–30 kV) apparatus from Hitachi (Tokyo, Japan), and the dispersion state of the rubber phases into ABS and TPO filaments was evaluated. All the samples were previously cryofractured and coated using a gold sputtering technique to avoid any charging effect during the electron beam scanning.

#### 2.3.3. Thermogravimetric Analysis (TGA)

TGA analysis was carried out by using TGA Q50 device from TA Instruments, while applying a temperature ramp from ambient to 700 °C (heating rate of 10 °C/min) under nitrogen flow of 60 mL/min and sample wight of 10 mg.

#### 2.3.4. Melt Flow Index Characterization (MFI)

Melt flow index tester MFI-10 Davenport from AMETEK (Largo, FL, USA) was used to evaluate the effect of recycled tire powders, in the presence or absence of SEBS, on the fluidity of TPO-based materials at 200 °C and under a load of 5 kg. The material weight that flowed through the cavity for 10 min gave an MFI of g/10 min.

## 3. Results

### 3.1. ABS-Based Composites

#### 3.1.1. TGA Analysis

**Figure 4:**
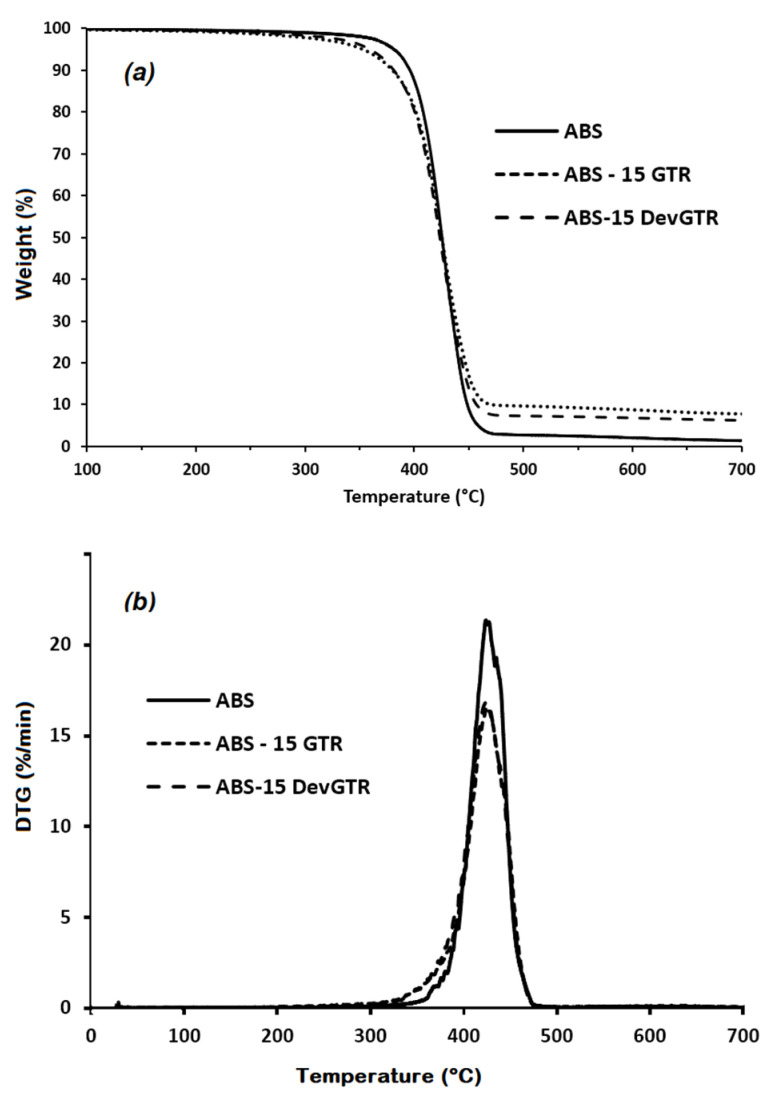
TGA (**a**) and DTG (**b**) thermograms of ABS-based composites (under N_2_, 10 °C/min).

The thermal stability of GTR- and DevGTR-loaded ABS composites were evaluated by TGA analysis. Based on the presented TGA and DTG thermograms (Figure 4), it was found that the tire microparticles, independently of their nature, induced a slight premature thermal degradation of the polymer composites (−4%) in comparison to the pristine ABS (−2%) at 350 °C. Since this mass loss is very limited, we assume that the weight loss obtained for the composite is the addition of the decomposition of both phases separately. No further decomposition induced by one phase on the other occurs. The main thermal decomposition step of the composites remains similar to that of pristine ABS, and takes place at 422 °C, as can be clearly evidenced by DTG thermograms. It was also noticed that the residue obtained at the end of the thermal decomposition of the ABS-based composites corresponded to the mineral fraction presented in the tire rubber fraction.

#### 3.1.2. Mechanical Properties

First, we start by identifying the optimal 3D printing parameters for the pristine ABS. An overview of the obtained mechanical properties is presented in Table 3 relative to the mechanical properties of the ABS specimens prepared by injection molding and the 3D-printed samples based on two different raster angles, i.e., 45°/−45° and 90°/0°. Based on the obtained data, the raster angle of 45°/−45° was selected as a printing pattern for the rest of the study, since it enables the production of materials with higher impact resistance compared to the 90°/0° raster samples. Specimens printed with 90°/0° raster presented a complete break during impact test, while those prepared at 45°/−45° had similar behavior to ABS and did not break completely.

The incorporation of the GTR, independently of the processing method (injection molding vs. 3D printing) and the rubber content (15 wt.% and 30 wt.%), did not lead to an enhancement of the ABS composites mechanical properties (Table 4). Furthermore, the incorporation of GTR, even at only 15 wt.% loading rate, induced some significant decrease of the ABS impact resistance, regardless of the nature of the process used for the specimen preparation. In fact, the impact resistance of ABS loaded with 15 wt.% GTR decreased to 3.7 kJ/m^2^, when the materials were injected/molded and to 3.4 kJ/m^2^ when they were prepared by 3D printing. In the case of the composites containing 30 wt.% GTR, the decrease in the recorded values was even more important, since the impact resistance decreased to 2.8 kJ/m^2^, again independently of the process used. These observations were accompanied by low Young’s modulus values, as a result of the incorporation of a higher fraction of the soft recycled rubber. 

**Figure 5:**
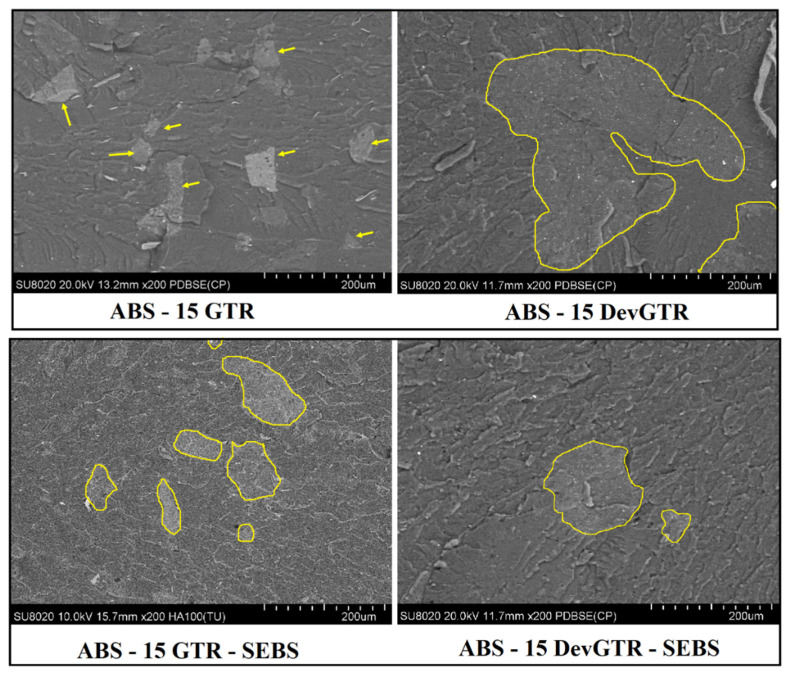
SEM micrographs of ABS-based materials containing GTR or DevGTR with or without the addition of SEBS.

In conclusion, the incorporation of recycled tire microparticles induced a drop in the material mechanical properties, despite the affinity existing between both phases leading to a fine dispersion state of GTR domains, and the good wettability of the rubber by the polymeric matrix (absence of voids or cracks), as evidenced in the SEM micrographs (Figure 5, see ABS-15 GTR).

However, the obtained results were not of significant importance since ABS is considered a technical polymer, demonstrating high mechanical performance and the presence of the rubber fraction known as inert fillers often responsible for a deterioration of the composite mechanical properties, usually explained by the weak interfacial interaction between the rubber and thermoplastic phases [16,17,18]. As an alternative approach, it is of interest to add an interfacial compatibilizer aiming at enhancing the interactions between polymer matrix and the rubber domains and reducing the interfacial energy between the components.

Based on these results, the content of the incorporated recycled tire into ABS was limited to 15 wt.%, while SEBS was added as compatibilizing agent. Furthermore, GTR rubber waste was replaced by DevGTR, thus a more malleable and deformable form of rubber waste triggered by chemically devulcanization. The final composite materials were thus produced by FDM 3D printing. DevGTR presents an interesting advantage owing to its ability to melt and flow during melt processing, as clearly evidenced by SEM (Figure 5, see ABS-15 DevGTR). 

First, and in the absence of SEBS, replacement of GTR by DevGTR allows us to enable some enhancement of the composite mechanical properties in comparison with the ABS-GTR composites. The impact resistance increased from 3.4 kJ/m^2^ for ABS-15 GTR to 4.4 kJ/m^2^ for ABS-15 DevGTR. However, this value still remained relatively low, the specimens breaking down completely during the impact tests. This slight improvement of the impact resistance was obtained despite the presence of dispersed rubber domains presenting larger average size compared to the initial DevGTR particles (Figure 5, ABS-15 DevGTR). The formation of such large rubber domains results from the coalescence of the devulcanized rubber particles during melt processing, which allows the blend to reduce its total free energy through phase separation as it is commonly observed in the case of immiscible polymer blends [19,20].

**Figure 6:**
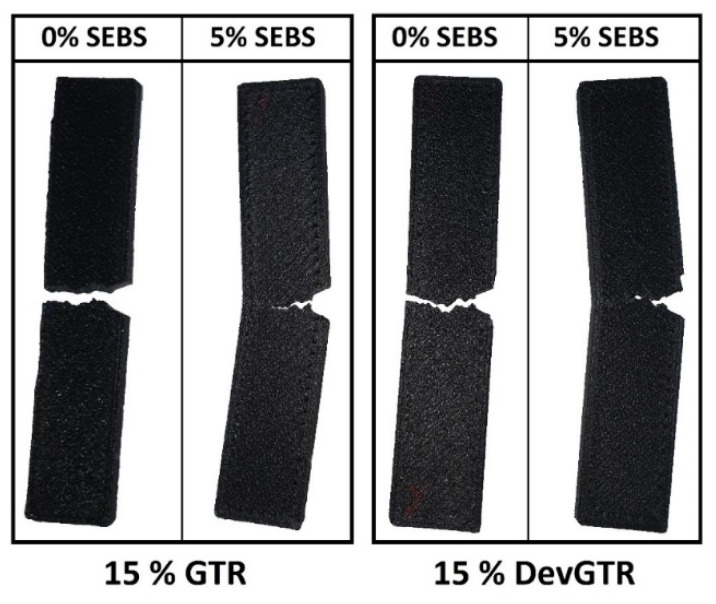
Images of ABS impact specimens (after test), containing 15 wt.% GTR or DevGTR, with and without the incorporation of SEBS (5 wt.%).

As expected, SEM micrographs (Figure 5; see ABS-15 GTR and ABS-15 DevGTR) highlight the effect of SEBS as a compatibilizer. Addition of SEBS (via the partial substitution of only 5 wt.% ABS) allowed for decreasing the interfacial tension between the two immiscible phases (comparison between micrographs ABS-15 GTR vs. ABS-15-GTR-SEBS; Figure 5) and acting as barrier against coalescence (comparison between micrographs ABS-15 DevGTR vs. ABS-15-DevGTR-SEBS; Figure 5). These morphological modifications triggered by the presence of SEBS compatiblizer are further confirmed by the incomplete break upon impact test (as compared to the previously observed complete fracture in the absence of SEBS) (Figure 6). These results again demonstrate the efficiency of SEBS for compatibizing ABS and recycled tire rubber, likely induced by the presence of styrene groups as aforementioned in the Introduction section and reported in the literature. 

For all investigated blends, the presence of recycled tire rubber (GTR or DevGTR) led to a slight reduction of materials’ stiffness (Young’s modulus). Actually, this evolution was expected due to the presence of the rubber phase conferring a certain degree of softness to the composite material.

With this, we demonstrated that combining recycled tire rubber and SEBS presents an interesting solution for the enhancement of ABS-based materials’ mechanical properties, where the rubber content was fixed at 15 wt.%. In addition, the incorporation of GTR seems to be the best approach for the valorization of recycled tire rubber, since it requires less preparation than chemically devulcanized rubber. Such formulations could present some interest, especially for recycled ABS materials that are known to lose impact resistance along the materials ageing process [21], often resulting in oxidation and partial crosslinking of polybutadiene nodules induced by UV irradiation [22,23,24].

### 3.2. TPO-Based Composites

#### 3.2.1. TGA Analysis

**Figure 7:**
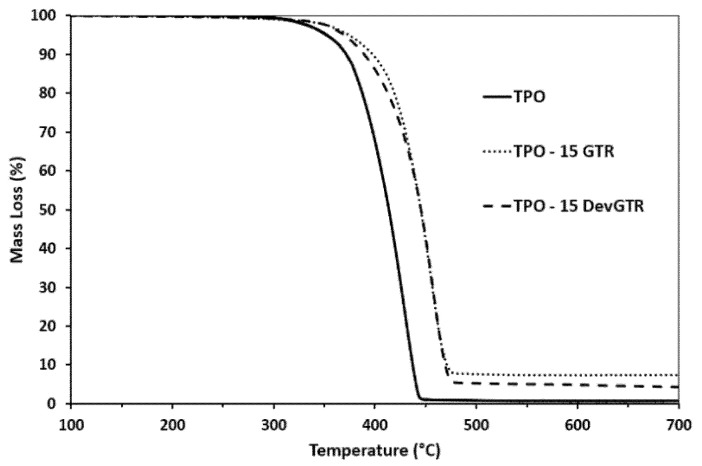
TGA thermograms of TPO, TPO-GTR, and TPO-DevGTR materials (under N_2_, 10 °C/min).

The impact of the incorporated recycled tire microparticles onto the TPO-based composites’ thermal degradation was evaluated by TGA analysis. The TGA thermograms of the pristine TPO and related composites containing 15 wt.% GTR and DevGTR are presented in Figure 7. It was evidenced that the thermal stability of the composites became higher than the pristine TPO, even if both recycled tire powders presented lower thermal stability than the pristine TPO. In fact, the temperature at a weight loss of 5% (T-5%, Figure 7), for both GTR and DevGTR powders, was 230 °C. In contrast, the TPO one was 353 °C and increased up to 370 °C when 15 wt.% of GTR and DevGTR were loaded. This result is of particular interest and revealed that the incorporation of recycled tire powder (GTR or DevGTR) did not lead to any premature TPO thermal degradation and in contrast allowed the production of thermally stable TPO-tire loaded composites.

#### 3.2.2. MFI Analysis

The effect of the recycled tire powders, in the presence or absence of SEBS, on the molten viscosity of TPO-based materials, was evaluated by measuring the MFI (200 °C/5 kg). The obtained results are summarized in Table 5. It was found that the incorporation of 15 wt.% recycled tire (GTR or DEVGTR) led to a slight increase in the composite viscosity; the MFI values decreased from 9.8 g/10 min for pristine TPO to 8 and 8.7 g/10 min for TPO containing GTR and DevGTR, respectively. This evolution is commonly observed for polymeric materials containing inorganic fillers. Furthermore, an additional decrease in the MFI values was obtained, once the compatibilizing agent was added to the blend. In fact, MFI decreased to 7.4 g/10 min and 7.1 g/10 min for TPO containing 5 wt.% SEBS in combination with 15 wt.% GTR or DevGTR, respectively. The observed increase in the viscosity values, in presence of the SEBS, presents another manifestation of the compatibilizing effect of SEBS, which is located between TPO and rubber phases and promotes the adhesion between both phases. Similar behavior (increase in the viscosity) after the addition of different compatibilizing agents (SEBS, SEBS-*g*-maleic anhydride, polypropylene-*g*-maleic anhydride) has been already reported in the literature and attributed to the enhancement of the interfacial adhesion between the polymer and rubber phases [25,26].

#### 3.2.3. Mechanical Properties

**Figure 8:**
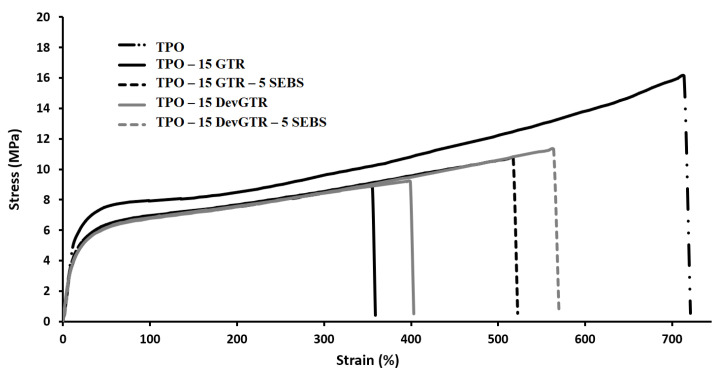
Stress–strain curves of TPO and TPO-recycled tire rubber blends as prepared by 3D printing.

GTR and DevGTR powders were incorporated into TPO by Brabender internal mixer, and the obtained materials were used for the preparation of filaments suitable for 3D printing. For the sake of comparison, production of specimens by compression molding has been carried out as well. The stress–strain graphs are presented in Figure 8, and the data are summarized in Table 6. It was found that the polymer processing procedure did not significantly impact the TPO mechanical properties (compression-molding vs. 3D printing). In addition, the materials presented high elongation at break (>700%) and Young’s modulus of 100 MPa, accompanied by a stress at break of 18 MPa and a break during any impact test.

However, the incorporation of 15 wt.% of GTR or DevGTR affected the mechanical properties of TPO at different levels. On one hand, GTR and the specimens processed by compression-molding presented limited reduction of the elongation at break, which decreased from 850% to 670%, while a significant reduction of this parameter was observed for the other samples: 370% for the 3D printed TPO-15 GTR, 206% for the compressed-molded TPO-15 DevGTR, and 545% for the 3D printed TPO-15 DevGTR. This was accompanied by a decrease in the Young’s modulus and the stress at break values. The drop in the ultimate mechanical properties is commonly obtained for non-compatibilized blends exhibiting low interfacial adhesion. However, it should be mentioned that none of the blends was broken during impact testing, and the specimens remained like their initial forms. This finding demonstrated that the incorporation of the rubber phase (GTR or DevGTR) within the TPO matrix did not affect the materials impact resistance, which remained governed by the TPO continuous phase.

**Figure 9:**
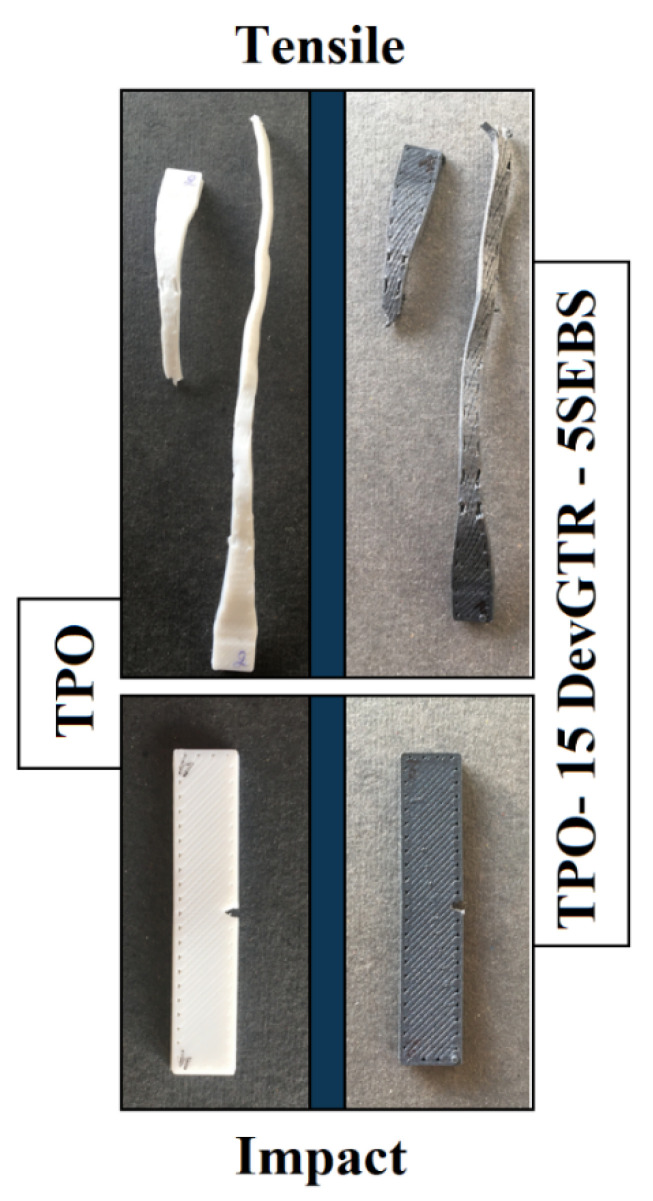
Images of 3D printed specimens after tensile and impact tests.

On the other hand, the incorporation of the compatibilizing agent enabled to a certain degree the enhancement of the material mechanical properties. A greater impact was noticed on the elongation at break that increased from 370% to 545% for the 3D-printed TPO-15 GTR-5 SEBS and from 400% to 550% for 3D-printed TPO-15 DevGTR-5 SEBS, indicating the improved adhesion between the phases. The general profile of the stress–strain curves remained like the pristine TPO (Figure 8), as well as the elongation and deformation upon traction of the specimens during both tensile and impact tests (Figure 9). The incorporation of SEBS within TPO containing 15 wt.% GTR did not induce any enhancement of elongation at break for compressed molded materials, while the positive effect of SEBS is clearly demonstrated for 3D printed materials.

**Figure 10:**
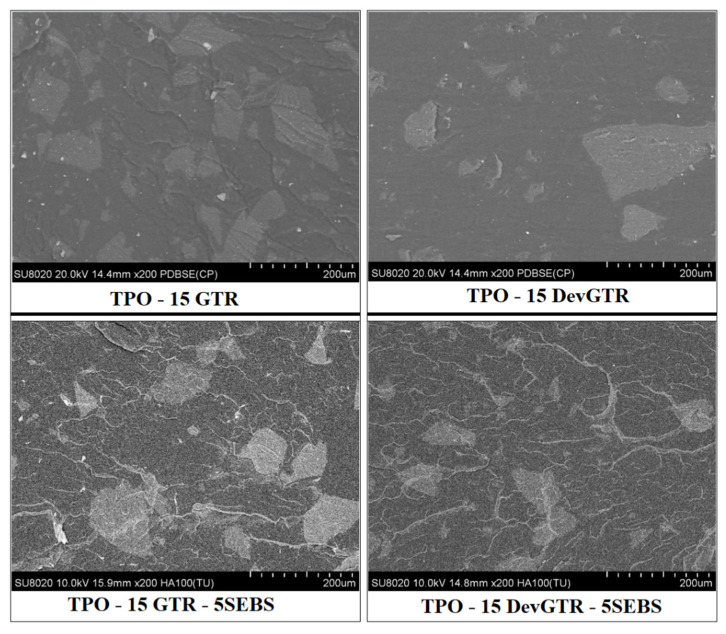
SEM micrographs of TPO containing 15 wt.% GTR or DevGTR, with or without the incorporation of 5 wt.% SEBS.

The present data confirmed the advantage of the SEBS incorporation and its positive impact on the interfacial adhesion between TPO and rubber phase, as was previously highlighted by MFI measurements (Table 5). Moreover, the compatibilization effect of SEBS favored the dispersion of rubber particles into the TPO matrix, especially in the case of TPO containing DevGTR microparticles, as demonstrated in Figure 10 and in agreement with the previous observations performed with the ABS matrix. SEM micrographs revealed again the good dispersion of DevGTR domains within the polymer matrix, as a result of the barrier effect of SEBS preventing the rubber particles coalescence and the formation of larger domains. This is due to the chemical interaction between styrene groups present in both SEBS and Recycled rubber (GTR and DevGTR), as well as to the physical entanglement between ethylene/butylene with TPO chains.

In these systems, the increase in the GTR content up to 30 wt.% led to a clear reduction in the composite ductility, which decreases to 265% for compressed molded specimens and 290% for 3D printed specimens. A similar evolution was obtained even in the presence of compatibilizing agent (5 wt.% SEBS, Table 6). However, the impact resistance of this composite remained high (no break during impact test) for all compositions.

**Figure 11:**
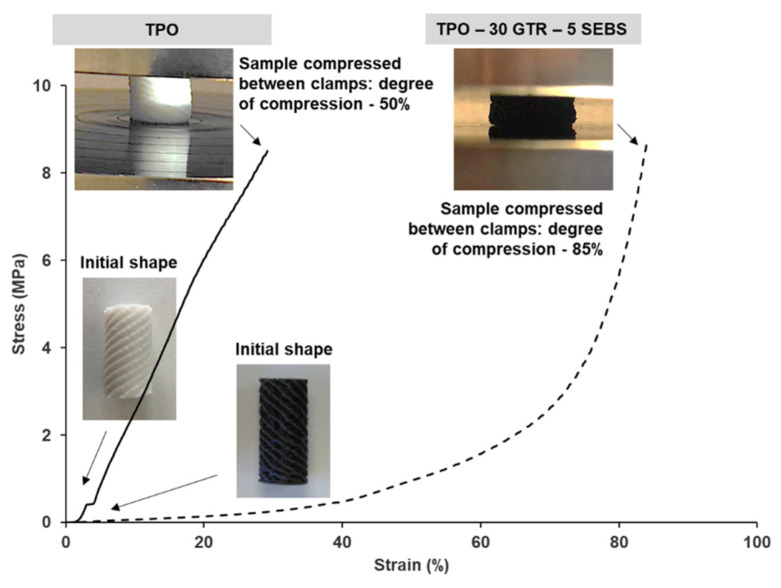
Stress–strain compression curves for TPO and TPO-30 GTR-5 SEBS samples.

**Figure 12:**
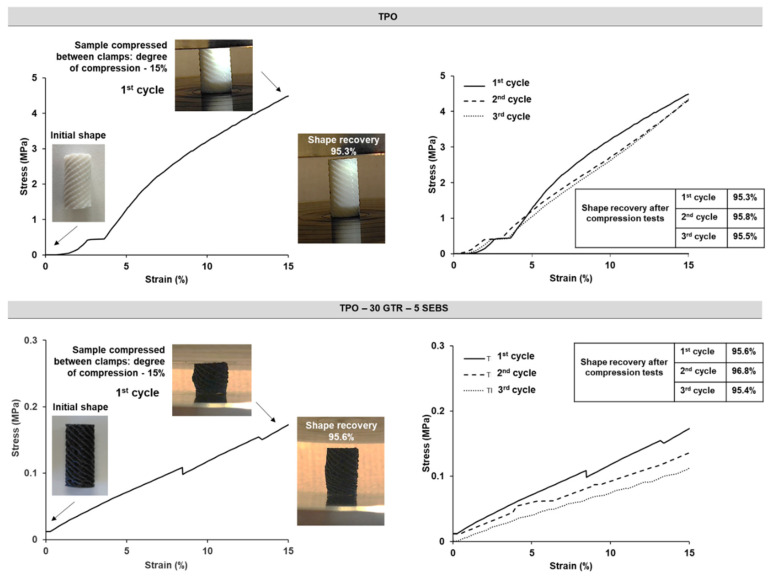
Stress–strain compression curves: first cycle of compression and digital images of the samples (on the left) and reproducibility of the compression tests (three successive fatigue cycle tests; on the right) of TPO and TPO-30 GTR-5 SEBS samples.

Interestingly, these compositions were also characterized by compression testing. As a first step, the materials were subjected to maximal compression, in order to define the maximal stress that the specimens could support before break. Both studied systems (TPO and TPO-30 GTR-5 SEBS) did not display any break during the tests: the TPO specimen slipped between the clamps upon compression greater than 12 MPa, while the TPO-30 GTR-5 SEBS supported high compression (85% of compression strain with compression stress of 8.5 MPa). As discussed above, the TPO materials were characterized by higher Young’s modulus values explaining the lower degree of strain compression (50%) at 8.5 MPa, compared to the TPO-30 GTR-5 SEBS (85%) (Figure 11). The higher compression strain of the GTR-SEBS-containing samples was explained by the increased elastomeric properties of the materials, due to successful incorporation of the high fraction in recycled tire rubber, i.e., 30 wt.%. Of interest was to define the material shape recovery upon strain defining their future functional performances. For this purpose, three successive compression tests were done on the same sample, while reaching 15% compression at each compression cycle (Figure 12). Higher compressive strength was observed for the TPO samples (4.3 ± 0.02 MPa), while for the TPO-30 GTR-5 SEBS, this parameter was lower (0.13 ± 0.1 MPa). The obtained results were in accordance with the literature, where ABS-based FDM-printed samples presented no break upon compression and lower compressive strength due to the rubber content, for instance, in comparison to PLA and PLA-based composites [27]. In addition, it was found that all the samples presented good shape recovery, with shape recovery ratio (Rr) between 95% and 97% (Figure 12). The reproducibility of the results was confirmed, revealing preservation of the blend material mechanical properties upon compression (three successive compression cycles, up to 15% of compression strain). The good compression behavior of the specimens demonstrated the potential application of the materials, e.g., as resorts, joints, or impact absorbing parts.

## 4. Conclusions

In the present study, devulcanized or vulcanized recycled tire rubber (DevGTR or GTR) was incorporated into ABS or TPO polymer matrices at different content, i.e., 15 wt.% or 30 wt.%, for the production of the filaments suitable for 3D printing by FDM. The printed materials were fully characterized, and their performances were compared with injected or thermo-compressed specimens similar in chemical composition. Within the studied systems, use of SEBS as a compatibilizing agent was found to be recommended for obtaining composite materials with superior mechanical performances. Owing to olefin and styrene segments present, respectively, in both polymeric matrices, the incorporation of SEBS allows for the interfacial tension between the two immiscible tire and polymeric phases to be decreased and acts as barrier against the coalescence of DevGTR particles.

TPO-based composites proved to be of particular interest for the good dispersion of the rubber particles within the polymer matrix and their good impact and compression properties, accompanied with high shape-recovery ratio. In particular, the compatiblized TPO-GTR-SEBS materials displayed good potential as shock-absorbing part applications (e.g., resorts, mechanical pieces or joints).

In ABS-based systems, the combination of recycled tire rubber with SEBS is required prior obtaining materials with enhanced impact resistance. This kind of combination seems adapted for recycled ABS materials that are known to lose impact resistance in the aging processes of materials. With the present work, the authors hope to open up new possibilities for improving the circular economy of the automotive industry through the recovery of recycled tire rubber from automotive waste and combining it with polymer matrices such as ABS and TPO for their wide use in the sector.

## Figures and Tables

**Table 1:** Filament manufacturing settings. The direction of extrusion is set from H4 to H1.

Matrix	Heater 4 (°C)	Heater 3 (°C)	Heater 2 (°C)	Heater 1 (°C)	Screw Speed (rpm)	Fan Speed (%)
ABS	230	220	210	200	7	100
TPO	210	205	200	195	5	30

**Table 2:** Main printing parameters used for the preparation of the 3D printed specimens.

Matrix	Extruder Temperature (°C)	Plate Temperature (°C)	Box Temperature (°C)	Extrusion Width (mm)	Layer Thickness (mm)	Nb Loops	Infill (%)	Flow (mm^3^/s)	Printer Speed (mm/s)	Raster Angle (degree)
ABS	245	110	80	0.4	0.2 (1^st^ = 0.3)	2	100	0.8–6	25	90/0 and 45/−45
TPO	245	60	25	1	0.2 (1^st^ = 0.3)	1	100	4–10	5	45/−45

**Table 3:** Mechanical properties (tensile and impact tests) of pristine ABS specimens prepared by injection molding or 3D printing using two raster angles (90°/0° and 45°/−45°).

By Injection Molding			By 3D Printing					
Young’s Modulus (MPa)	Strain at Break (%)	Impact Resistance (kJ/m^2^)	Young’s Modulus (MPa)		Strain at Break (%)		Impact Resistance (kJ/m^2^)	
2264 ± 115	10.7 ± 4.3	Partial	90°/0°	45°/−45°	90°/0°	45°/−45°	90°/0°	45°/−45°
			1737 ± 55	1840 ± 100	5.2 ± 0.5	6.7 ± 1.2	6.9 ± 0.8	Partial

**Table 4:** Mechanical properties (tensile and impact tests) of ABS-based composites loaded with recycled GTR or DevGTR, containing or not compatibilizing agent-SEBS.

	by Injection Molding			by 3D Printing		
Composition	Young’s Modulus (MPa)	Strain at Break (%)	Impact Resistance (kJ/m^2^)	Young’s Modulus (MPa)	Strain at Break (%)	Impact Resistance (kJ/m^2^)
ABS	2264 ± 115	10.7 ± 4.3	Partial	1840 ± 100	6.7 ± 1.2	Partial
ABS-15 GTR	1930 ± 80	5.7 ± 0.4	3.7 ± 0.3	1190 ± 60	3.6 ± 0.6	3.4 ± 0.4
ABS-30 GTR	1660 ± 70	5.5 ± 0.4	2.8 ± 0.1	1046 ± 22	4 ± 0.5	2.8 ± 0.3
ABS-15 GTR-5 SEBS	n.a.	n.a.	n.a.	1130 ± 50	4.8 ± 0.4	Partial
ABS-15 DevGTR	n.a.	n.a.	n.a.	1110 ± 40	5 ± 0.5	4.45 ± 0.1
ABS-15 DevGTR-5 SEBS	n.a.	n.a.	n.a.	1130 ± 30	3.8 ± 0.4	Partial

**Table 5:** MFI values of pristine TPO, TPO-GTR, and TPO-DevGTR materials (200 °C/5 kg).

Matrix	MFI (g/10 min)
TPO	9.8 ± 0.4
TPO-15 GTR	8.0 ± 0.2
TPO-15 GTR-5 SEBS	7.4 ± 0.2
TPO-15 DevGTR	8.7 ± 0.2
TPO-15 DevGTR-5 SEBS	7.1 ± 0.1

**Table 6:** Mechanical properties (tensile and impact tests) of TPO-based composites loaded with recycled GTR or DevGTR, containing or not SEBS.

	by Thermo-Compressing			by 3D Printing		
Composition	Young’s Modulus (MPa)	Strain at Break (%)	Impact Resistance (kJ/m^2^)	Young’s Modulus (MPa)	Strain at Break (%)	Impact Resistance (kJ/m^2^)
TPO	100 ± 12	850 ± 100	No break	93 ± 5	740 ± 40	No break
TPO-15 GTR	76 ± 10	670 ± 80	No break	64 ± 4	370 ± 25	No break
TPO-30 GTR	68 ± 12	265 ± 60	No break	40 ± 5	290 ± 40	No break
TPO-15 GTR-5 SEBS	88 ± 4	360 ± 15	No break	71 ± 2	545 ± 50	No break
TPO-30 GTR-5 SEBS	60 ± 3	360 ± 60	No break	25 ± 2	233 ± 40	No break
TPO-15 DevGTR	70 ± 6	206 ± 115	No break	63 ± 4	400 ± 20	No break
TPO-15 DevGTR-5 SEBS	52 ± 3	510 ± 100	No break	66 ± 2	550 ± 60	No break

## Data Availability

Not applicable.
